# Importance of STAT3 signaling in preeclampsia (Review)

**DOI:** 10.3892/ijmm.2025.5499

**Published:** 2025-02-06

**Authors:** Daniela Marzioni, Federica Piani, Nicoletta Di Simone, Stefano Raffaele Giannubilo, Andrea Ciavattini, Giovanni Tossetta

**Affiliations:** 1 Department of Experimental and Clinical Medicine, Polytechnic University of Marche, I-60126 Ancona, Italy; 2 Hypertension and Cardiovascular Risk Research Center, Department of Medical and Surgical Sciences, Alma Mater Studiorum University of Bologna, I-40126 Bologna, Italy; 3 Department of Biomedical Sciences, Humanitas University, I-20072 Milan, Italy; 4 Scientific Institutes for Hospitalization and Care (IRCCS), Humanitas Research Hospital, I-20089 Rozzano, Italy; 5 Department of Clinical Sciences, Polytechnic University of Marche, I-60123 Ancona, Italy

**Keywords:** STAT3, preeclampsia, compound, hypoxia, inflammation

## Abstract

Placentation is a key process that is tightly regulated that ensures the normal placenta and fetal development. Preeclampsia (PE) is a hypertensive pregnancy-associated disorder characterized by increased oxidative stress and inflammation. STAT3 signaling plays a key role in modulating important processes such as cell proliferation, differentiation, invasion and apoptosis. The present review aimed to analyse the role of STAT3 signaling in PE pregnancies, discuss the main natural and synthetic compounds involved in modulation of this signaling both *in vivo* and *in vitro* and summarize the main cellular modulators of this signaling to identify possible therapeutic targets and treatments to improve the outcome of PE pregnancies.

## 1. Introduction

The placenta is a transitory but key organ that undergoes changes during pregnancy ensuring the normal fetal development (1,2). Placental development is a process tightly regulated during pregnancy and its alteration can lead to complications such as preeclampsia (PE) (3), fetal growth restriction (FGR) (4), gestational trophoblastic disease (5), preterm delivery (6) and gestational diabetes mellitus (GDM) (7). In addition, placental development is impaired by exposure to exogenous agents such as bacteria (8), viruses (9) and pollutants (10,11) that can alter the normal placental function.

Preeclampsia (PE) is a hypertensive disorder of pregnancy with incidence between 2 and 10% of pregnancies worldwide (12). It is generally diagnosed from the second trimester of gestation and is clinically characterized by *de novo* maternal hypertension (diastolic blood pressure of 90 mmHg and/or systolic blood pressure of 140 mmHg) and proteinuria (>300 mg/24 h) (13,14). A high body mass index, previous preeclamptic pregnancy, advanced maternal age and nulliparity are important risk factors of PE (15,16).

Although clinical diagnosis of PE occurs after 20 weeks of pregnancy, it is hypothesized that placental impairment begins during early stage of pregnancy and may be due to poor trophoblast invasion (12). Poor endometrial invasion of the extravillous trophoblast (EVT) into the maternal uterine wall impairs proper remodelling of endometrium spiral arteries, altering vascular perfusion and leading to a hypoxic environment. This causes increased oxidative stress and inflammation that leads to trophoblast immaturity and altered angiogenesis of placental villi (17,18).

Depending on the gestational age of occurrence of clinical signs and symptoms, PE is divided into late-(≥34 weeks of gestation) and early-onset PE (<34 weeks of gestation). Late-onset PE accounts for the majority of preeclampsia cases (~90%), while early-onset PE is less common but associated with higher rates of neonatal mortality and a greater degree of maternal morbidity compared with late-onset PE (19,20).

Late-onset PE is a serious condition since it can lead to eclampsia and hemolysis, elevated liver enzyme and low platelet syndrome (20,21). Early-onset PE is associated with impaired remodelling of the uterine spiral arteries, which leads to hypoxia, trophoblast immaturity, maternal systemic inflammation, vascular dysfunction and FGR (18,22,23) while late-onset PE is associated with maternal endothelial dysfunction (19,22). Both late- and early-onset PE show increased oxidative stress and inflammatory response that can cause maternal and fetal complications (19,20,24). Thus, early- and late-onset PE are considered distinct forms of PE with different pathophysiology and pregnancy outcomes.

**Figure 1:**
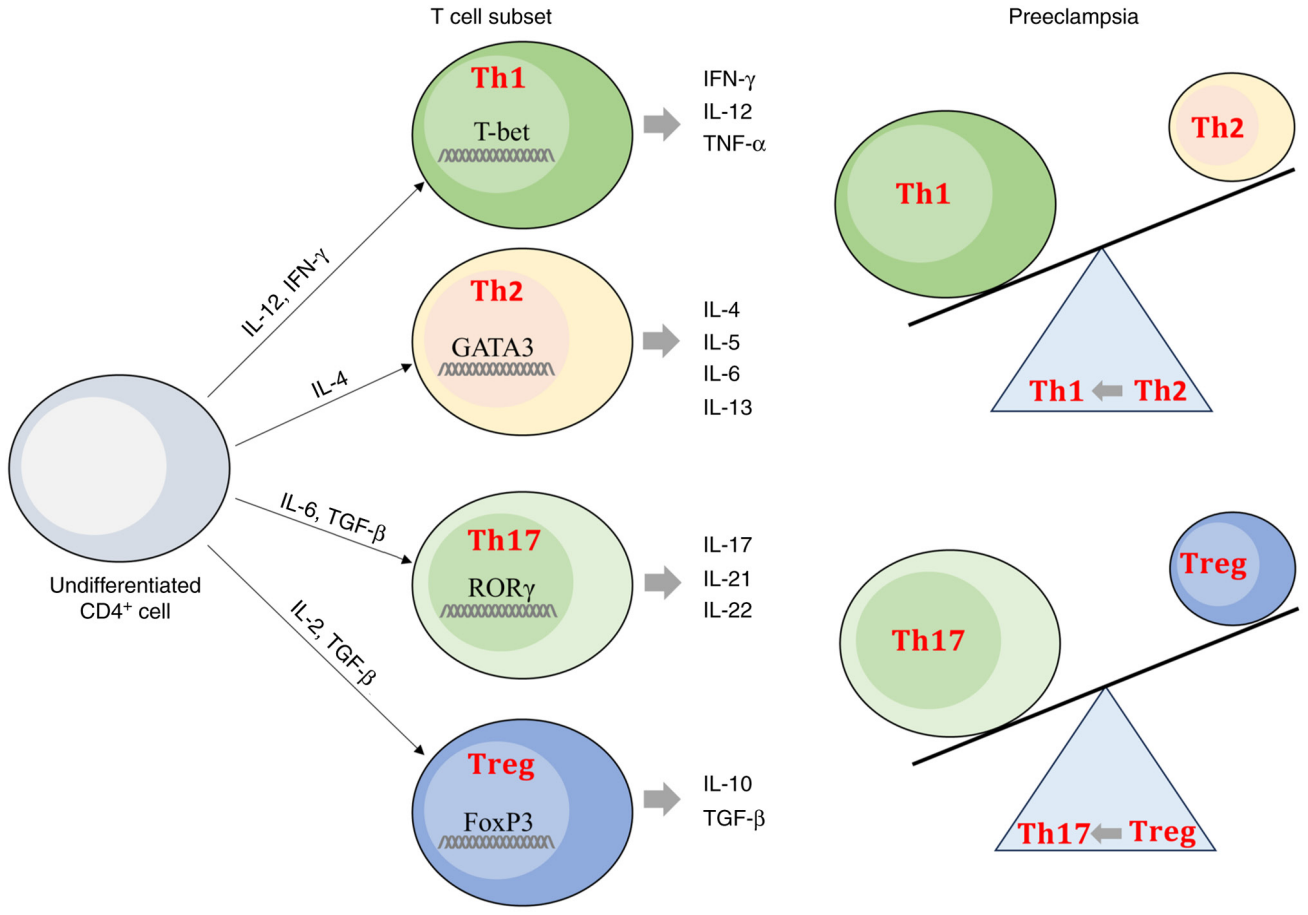
T cell subset differentiation. Cytokine-dependent differentiation of CD4^+^ T cells in Th1, Th2, Th17 and Treg cells is unbalanced in preeclampsia. Treg, regulatory T cell; Th, T helper cell; FoxP3, forkhead box P3; ROR, tyrosine kinase-like orphan receptor; T-bet, T-box protein.

Numerous studies (25-27) have observed involvement of the immune system in the development of PE. Moreover, the cytokine environment serves a pivotal role in the differentiation of T cell subsets (Fig. 1). T helper (Th) 1 cells are induced by IFN-γ and IL-12, two inflammatory mediators involved in the activation of the adaptive immune response (28). Th2 cell differentiation is induced by IL-4 while Th17 cell differentiation is induced in presence of IL-6 and transforming growth factor-β (TGF-β). TGF-β is also responsible for T regulatory (Treg) cell differentiation, an important T cell subset in maintaining self-tolerance and pregnancy (29,30). Th17 cells are considered the key effector T cells in induction of the inflammatory response (28,30).

PE pregnancies show an imbalance between inflammatory Th1/Th17/Th2 and Treg profiles (30,31). In PE pregnancies, CD4^+^ T cell subsets have a high expression of transcription factor T box (T-bet) and retinoic acid receptor-related orphan receptor γ (RORγt), which are characteristic of Th1 and Th17 profiles, respectively, and decreased expression of GATA 3 binding protein (GATA-3), and forkhead box P3 (FoxP3), associated with Th2 and Treg profiles, respectively (32). Thus, PE pregnancies have a higher percentage of Th17 cells, while levels of Treg cells are lower, suggesting a shift from Treg toward Th17 cells in PE (33). PE pregnancies are also characterized by a shift from Th2 cells toward Th1 cells (34). An imbalance of T cell subsets can alter the immune microenvironment, leading to pregnancy complications, including PE (30,33).

Signal transducer and activator of transcription 3 (STAT3) is a signal transducer protein activated by the binding of cytokine or growth factors to their specific receptors. STAT3 serves a key role in regulating immune response by modulating Th1/Th17/Th2 and Treg gene expression profiles (35).

STAT3 is key for embryonic development: STAT3-deficient embryos die *in utero*; transient suppression of STAT3 notably decreases implantation and suppresses decidualization, demonstrating a key role of STAT3 in embryo implantation and development (36). Moreover, STAT3 regulates important trophoblast cell processes such as proliferation, migration, differentiation and apoptosis (37,38). Since these processes are altered in PE pregnancies (18,39-41), STAT3 signaling may play a key role in this pathology.

The aim of the present review is to provide an overview of the role of STAT3 signaling in PE pregnancy and understand the potential therapeutic use of STAT3 modulators to provide new therapeutic approaches in treatment and management of PE.

## 2. STAT3 signaling

**Figure 2:**
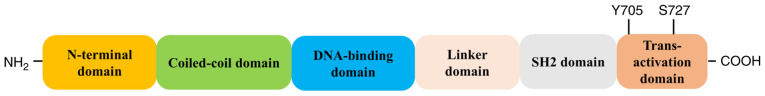
STAT3 domains. The SH2 domain regulates STAT3 dimerization while the trans-activation domain contains two phosphorylation sites: Tyrosine residue (Y) at 705 and a serine residue (S) at 727.

STAT family of proteins comprises seven transcription factors: STAT1, STAT2, STAT3, STAT4, STAT5a, STAT5b and STAT6. STAT3 has 770 amino acids and is organized in six domains (Fig. 2). The N-terminal domain is involved in interaction with other co-activators [such as c-Jun and CREB binding protein (CBP) p300]; the coiled-coil domain is necessary for the binding to the activated receptor, the DNA-binding domain is involved in recognizing the target DNA consensus sequence, the SH2 domain is necessary for STAT3 dimerization and recognizes the phosphorylated (p) tyrosine motifs (Y705 residue) in the STAT3 trans-activation domain (which contains conserved Tyr and Ser residues). STAT family members can form heterodimers (with another member of the STAT protein family) or homodimers (with another identical STAT protein) to initiate transcriptional activity of STAT-dependent genes following ligand stimulation (42,43).

When cytokines or growth factors bind to cell membrane receptors, STAT3 protein is activated (phosphorylated) by Janus kinase (JAK) family proteins, which are receptor-associated tyrosine kinases. Following phosphorylation, STAT3 forms a homodimer that is transferred into the nucleus via the nuclear pore complex (a GTP-dependent process) to bind the base sequence TTCnnGAA in the promoter of STAT3-dependent genes, thereby activating their transcription (44). STAT3 is also phosphorylated by non-receptor tyrosine kinases such as SRC and ABL (45,46).

**Figure 3:**
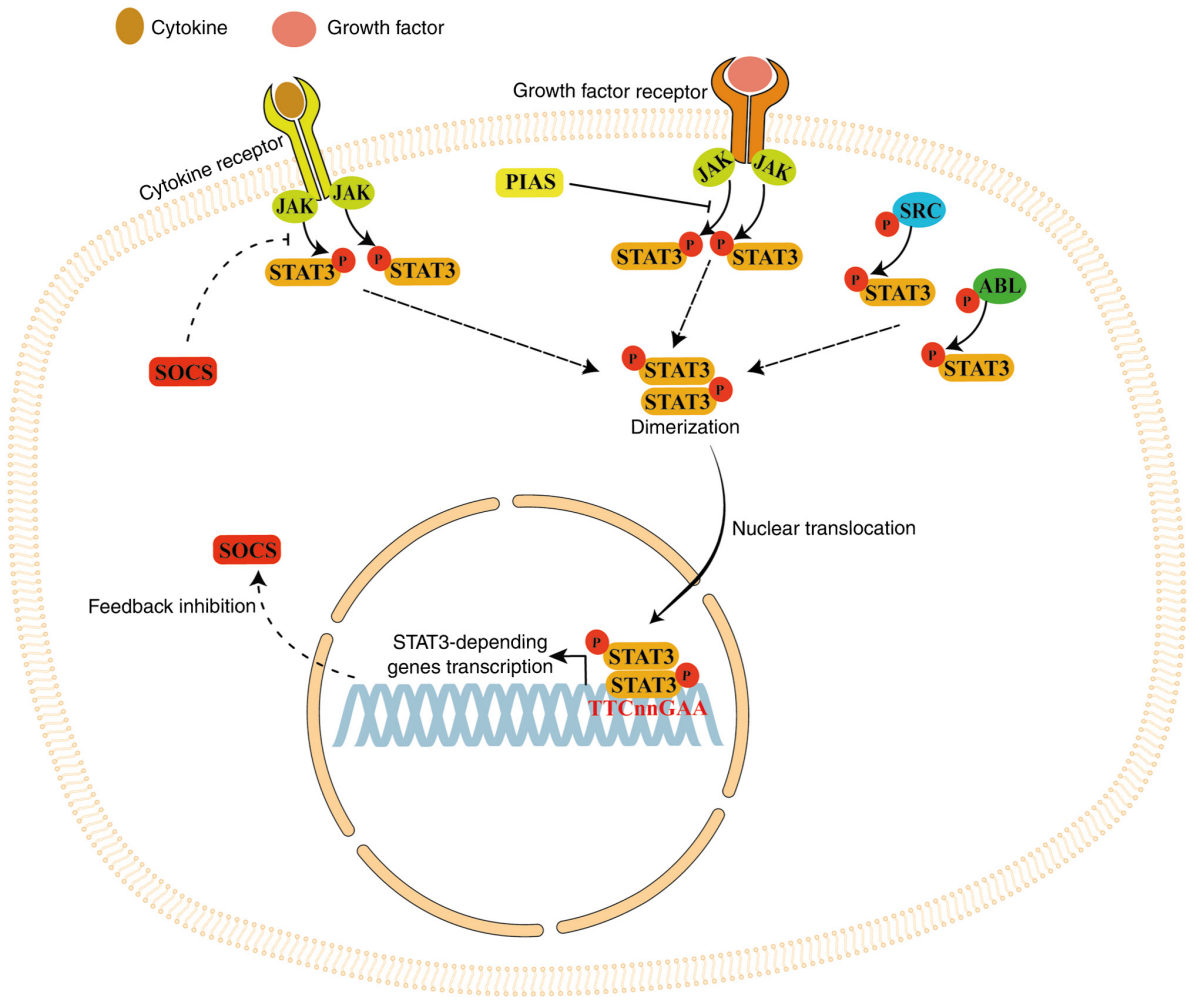
STAT3 signaling. When cytokines or growth factors bind their respective cell membrane receptors, STAT3 protein is phosphorylated by the receptor-associated tyrosine kinase JAK. Once phosphorylated, STAT3 forms a homodimer that is transferred into the nucleus to bind the base sequence TTCnnGAA in the promoter of STAT3-dependent genes, activating their transcription. STAT3 is also phosphorylated by non-receptor tyrosine kinases such as SRC and ABL. STAT3 long-term activation is inhibited by a negative feedback modulated by SOCS (which blocks STAT3 phosphorylation) and PIAS3 (which blocks the DNA-binding activity of STAT3). JAK, Janus kinase; SOCS, suppressor of cytokine signaling; PIAS3, protein inhibitor of activated STAT.

STAT3 long-term activation is inhibited by suppressors of cytokine signaling (SOCS) protein, creating a negative feedback loop (44). STAT3 signaling is also inhibited by the protein inhibitor of activated STAT, which blocks the DNA-binding activity of STAT3, inhibiting its transcriptional activity (44). STAT3 signaling serves a key role in several processes including cell proliferation, migration, survival and differentiation (Fig. 3) (42,44,47).

## 3. STAT3 signaling in PE

STAT3 serves a key role in the transcriptional activation of several proteins involved in the regulation of numerous cell processes such as apoptosis, cell proliferation and invasion (48,49). In trophoblast cells, STAT3 inhibition induces apoptosis and decreases cell proliferation and invasion, key processes involved in normal placental development (50-52). Thus, decreased STAT3 expression may inhibit EVT invasion into the maternal uterine wall, causing shallow trophoblast invasion and placental malperfusion, which are characteristics of PE pregnancy. These effects of STAT3 on trophoblast cells may be due to modulation of MMP activity since it has been demonstrated that STAT3 activation inhibits tissue inhibitor of metalloproteinase (TIMP)-1 expression (51).

STAT3 and pSTAT3 expression is significantly lower in human PE placentas compared with normal placentas (53-60). Thus, decreased STAT3 expression and activation in PE placental tissues serve an important role in the pathogenesis of PE. However, *in vivo* models of PE show contrasting results: To the best of our knowledge, there is only one study that reflects results obtained in humans (61). Other studies showed an increased pSTAT3 expression in placentas of PE models (62-65) while another study showed no alteration in pSTAT3 expression (66). Thus, studies focused on the role and modulation of STAT3 in *in vivo* models of PE must always be assessed for pSTAT3 expression to reflect the data found in humans.

Soluble Flt-1 (sFlt-1), also called soluble vascular endothelial growth factor (VEGF) receptor 1, is a soluble form of the VEGF and placental growth factor (PLGF) receptor, and plays a key role in decreasing levels of free PLGF and VEGF, causing endothelial dysfunction (67). sFlt-1 levels are increased in PE and associated with PE severity (68,69). Another important anti-angiogenic factor is the soluble endoglin (sEng), a co-receptor for TGFβ-1 and TGFβ-3, produced by proteolytic cleavage of endoglin and involved in angiogenesis and endothelial cell differentiation. sEng serves a key role in PE pathogenesis since it decreases circulating levels of TGFβ, a key modulator of angiogenesis (70), inhibiting TGFβ pathway and leading to endothelial dysfunction (71). Although the placenta is the primary source of these circulating factors, it has been demonstrated that peripheral blood mononuclear cells (PBMCs) may be an additional source of sFlt-1 and sEng (72).

A previous study (73) showed that STAT3 mRNA and protein levels are increased in CD4^+^ and CD8^+^ T lymphocytes isolated from patients with PE compared with those from normal pregnancies. The aforementioned study found an increase in Th17 lymphocytes while Treg population was notably lower in PE, demonstrating an increased Th17/Treg ratio in PE. Furthermore, levels of cytokines (IL-17 and IL-22) and anti-angiogenic molecules such as sEng and sFlt-1 are increased in isolated CD4^+^ cells from patients with PE, suggesting a possible association with PE pathogenicity. STAT3 is required for the IL-17 production by Th17, a subtype of CD4^+^ T lymphocytes (74). Thus, CD4^+^ T cells could participate in production of antiangiogenic factors characterising PE pregnancy. In particular, STAT3 could participate in favoring hypertension development during PE by increasing IL-17 levels, which serves an important role in hypertension (75).

STAT3 signaling serves a key role in angiogenesis since the inhibition of STAT3 phosphorylation suppresses this process (76). Normally, pSTAT3 is strongly expressed in the endothelial cells of first and second trimester placentas but its expression notably decreases in the third trimester, when angiogenesis is completed (77). However, pSTAT3 expression is notably increased in endothelial cells of PE placentas compared with normotensive controls, suggesting an association between STAT3 activation and placental angiogenesis defects in PE (78).

Placental factors in maternal circulation cause systemic endothelial dysfunction in PE pregnancy, increasing the risk of cardiovascular disease (CVD) following delivery (79-81). Christensen *et al* (82) found that human umbilical vein endothelial cell treatment with serum from patients with PE notably decreases STAT3(Y705) phosphorylation compared with serum from uncomplicated pregnancy. Moreover, sera from patients with previous PE, current hypertension and carotid atherosclerotic plaques shows significantly lower STAT3(Y705) phosphorylation capabilities compared with healthy controls with previous uncomplicated pregnancies 8-18 years after delivery. Thus, decreased serum-induced endothelial STAT3(Y705) activation may play an important role in PE-associated endothelial dysfunction and reduced endothelial STAT3(Y705) phosphorylation and may increase post-preeclamptic CVD risk after delivery (82).

Decreased STAT3 expression in PE placentas may favor trophoblast apoptosis in the hypoxic environment at the beginning of placentation (50-52). Moreover, decreased expression of STAT3 is associated with reduced cell proliferation and invasion due to decreased activity of MMPs (51). Decreased STAT3 expression can inhibit EVT invasion into the maternal uterine wall, causing a shallow trophoblast invasion, which is one of the primary causes of PE occurrence (12). Since STAT3 expression is significantly increased in CD4^+^ and CD8^+^ T lymphocytes isolated from patients with PE (73), STAT3 pathway is also involved, at least in part, in the increased inflammatory cytokines found in serum of patients with PE (83). Therefore, investigating the effects of STAT3 signaling modulation in PE pregnancy is necessary to understand and treat this complication of pregnancy.

## 4. STAT3 modulation in PE by natural and synthetic compounds

### STAT3 modulation by natural compounds

Natural compounds, also known as phytotherapeutics, are biological substances found in several plants, bacteria, fungi and marine organisms and are used worldwide due to anti-inflammatory, antioxidant and anticancer effects (84-87).

Silibinin is a flavonolignan derived from milk thistle (*Silybum marianum*) with antioxidant and anticancer properties (88). Xu *et al* (89) demonstrated that silibinin treatment of PBMCs, isolated from patients with PE pregnancy, decreases STAT3/RORγt expression, which is involved in the regulation of Th17 inflammatory profiles. In addition, PBMCs treated with silibinin show lower concentrations of inflammatory cytokines such as TNF-α, IL-6, and IL-23 and higher levels of IL-10 and TGF-β. Since there is a shift from Treg cells toward Th17 cells in PE pregnancy (33), silibinin may be an important immunomodulatory compound able to regulate the Th17/Treg cell balance in PE, decreasing inflammation (90).

Paeonol is a natural compound extracted from *Cortex Moutan*, a plant used in Chinese medicine with anti-inflammatory, anticancer and antioxidant effects (91,92). Paeonol significantly attenuates the inflammatory response in the placenta of PE mouse model, decreasing mRNA levels of TNF-α, IL-6 and IFN-γ and increasing IL-4 mRNA levels. Furthermore, paeonol treatment inhibits phosphorylation of JAK2 and STAT3 in the placental tissues of PE mouse model (62). These effects are reversed by treatment with SC-39100, a JAK2/STAT3 pathway agonist, demonstrating that paeonol anti-inflammatory effects are due to inhibition of the JAK2/STAT3 signaling pathway (62).

Vitamin D is a pro-hormone primarily obtained from exposure of the skin to ultraviolet B radiation and can regulate inflammatory responses, favouring the shift from a pro-inflammatory to a tolerogenic immune response by modulating numerous cells of the immune system including CD4^+^ T cells (93-95). Vitamin D deficiency may contribute to PE onset, altering Th1/Th17 and Th2/Treg profiles (96). Ribeiro *et al* (97) found that plasma levels of vitamin D are lower in PE compared with normal pregnancies. Vitamin D treatment of CD4^+^ T cells isolated from PE decreases STAT1/STAT4/T-bet and STAT3/RORγt activation, while increasing STAT6/GATA-3 and STAT5/FoxP3 activation. Treatment of PBMCs isolated from patients with PE with vitamin D also decreases levels of IFNγ, TNFα, IL-17, IL-22, IL-23 and IL-6, while increasing IL-10 and TGF-β levels, suggesting an immunomodulatory effect of vitamin D on STAT signaling that favours the shift from Th1/Th17 to Th2/Treg profiles. Vitamin D treatment may exert a beneficial effect in ameliorating systemic inflammation characterising PE pregnancies (97).

### STAT3 modulation by synthetic compounds

Studies (98-100) have reported that STAT3 can also be regulated by synthetic compounds. SO_2_, a major air pollutant produced by industrial factories and vehicle exhaust, can lead to pregnancy complications such as stillbirth, preterm birth, GDM and PE (101-104). Treatment of Swan.71 trophoblast cells with SO_2_ derivatives significantly decreases cell migration and invasion, arrests the cell cycle at S/G2/M phase and induces apoptosis (105). Moreover, SO_2_ derivatives notably increase IL-1β and decrease IL-6 secretion and STAT3 phosphorylation, leading to decreased expression of MMP2 and MMP9; this indicates SO_2_ derivatives exert their toxic effects on trophoblast cells, inhibiting IL-6/STAT3 signaling, which plays a key role in regulating cell viability, invasion and migration (105).

Pravastatin is a statin normally used to treat CVD but it can also reduce cholesterol content, alleviate inflammation, decrease oxidative stress and regulate endothelial functions (106,107). Wang *et al* (63) found that the expression of serum IL-6 in PE rat model (obtained by deoxycorticosterone acetate injection) is markedly higher than in controls and significantly reduced in PE rats treated with pravastatin. Moreover, PE rats show increased expression of pSTAT3 compared with controls rats. However, pSTAT3 expression is significantly decreased in PE rats treated with pravastatin. The proliferation of rat trophoblast cells is significantly decreased in PE rats (due to increased apoptosis) compared with controls but significantly increased in PE rats treated with pravastatin (which reduced apoptosis), indicating that pravastatin can inhibit IL-6/STAT3 signaling, decreasing the apoptosis of trophoblast cells in PE rats (63). These data are in contrast with another study that evaluated the role of pravastatin in a PE-like mice model (obtained by adenoviral overexpression of sFlt-1), which showed high blood pressure, abnormal vascular reactivity and proteinuria similar to PE (108,109). However, pSTAT3 placental expression is significantly higher in PE-like mice treated with pravastatin compared with untreated and control (normal) groups, suggesting that pravastatin can prevent PE and modulate STAT3 activation, further validating a beneficial role of statins in preventing PE (66). The contrasting results may be due to the different PE models.

Sulfasalazine is an anti-inflammatory drug used to treat arthritis and inflammatory bowel disease (110,111). However, sulfasalazine decreases placental secretion of the anti-angiogenic factor sFlt-1, expression of which is regulated by the epidermal growth factor receptor (EGFR) signaling pathways (112). By using primary cytotrophoblast cells, Hastie *et al* (113) found that sulfasalazine decreases sFlt-1 secretion, downregulating EGFR expression. Additionally, sulfasalazine notably decreases protein expression of ERK1/2 and STAT3, which are key adaptor molecules downstream of EGFR. Thus, sulfasalazine decreases sFlt-1 secretion, downregulating EGFR/ERK and EGFR/STAT3 signaling (113).

Nω-nitro-L-arginine methyl ester (L-NAME) is a L-arginine analogue used as a nitric oxide synthase inhibitor to treat hypotension. Due to these effects, it is also used to establish the PE-like rat model (114). L-NAME administration causes a decreased expression of STAT3 and pSTAT3 in the placenta of PE rats, suggesting a role of STAT3 signaling in the development of PE (61). Rats treated with L-NAME are widely used as an *in vivo* model to study PE pathophysiology (115-118).

Montelukast is a drug used in chronic asthmatic patients planning for pregnancy and is safely used during pregnancy (119). Montelukast is a selective cysteinyl leukotriene receptor antagonist with antioxidant and anti-inflammatory effects (120,121). Abdelzaher *et al* (64) demonstrated that montelukast treatment decreases oxidative stress and expression of IL-6, TNF-α, pJAK2, and STAT3 in PE rats. Thus, montelukast exerts anti-inflammatory effects, suppressing the IL-6/JAK2/STAT3 signaling pathway in PE rats (Table I) (64).

## 5. STAT3 modulation in PE by non-coding (nc)RNAs

ncRNAs are functional RNA molecules without protein-coding abilities. The most studied ncRNAs include microRNA (miRNA or miR), long ncRNA (lncRNA) and circular RNA (circRNA) (122-124). ncRNAs regulate expression of genes involved in several cellular processes including cell proliferation, invasion and metabolism (122).

miRNAs are a group of endogenous small single-strand ncRNAs that exert multifaceted functions in numerous diseases (123-125). The miR-133 family (comprising miR-133a and miR-133b) can affect invasion, migration and proliferation of tumor cells (126) and plays a key role in pregnancy complications such as recurrent spontaneous abortion (127). Placental tissue of patients with PE shows high levels of miR-133b and decreased pJAK2 and pSTAT3 expression. In HTR8/SVneo cells, hypoxia induces miR-133b expression and decreases pJAK2 and pSTAT3 expression and trophoblast migration and invasion while increasing apoptosis, thereby proving that miR-133b may exert its functions by regulating the JAK2/STAT3 pathway (56). Thus, inhibiting miR-133b may improve oxidative stress injury (induced by hypoxia) to promote the migration and invasion of trophoblasts and suppress apoptosis by activating the JAK2/STAT3 pathway (56).

Another miRNA involved in STAT3 modulation and altered in PE is miR-125b. This miRNA is associated with extra-villous trophoblastic proliferation and invasion, and its expression is notably increased in PE (128). Serum levels of miR-125b are significantly increased in patients with PE compared with normal pregnancies (128). Moreover, high levels of miR-125b decrease HTR-8/SVneo cell proliferation, invasion and migration as well as expression of STAT3, pSTAT3 and SOCS3, demonstrating that STAT3 is a target gene of miR-125b; high levels of miR-125b inhibit STAT3 signaling, reducing migration and invasion of extra-villous trophoblast cells (129).

In addition to miRNAs, several studies have demonstrated a key role of lncRNAs in pregnancy complications such as PE (130) and GDM (131). Dendritic cells (DCs) serve a key role as primary antigen-presenting cells at the beginning of pregnancy and express lnc-DC, which induces DC differentiation, maturation and STAT3 phosphorylation (132). Pregnancy complications characterized by impaired trophoblast invasion such as PE exhibit excessive DC maturity (133). Moreover, it has been reported that expression of lnc-DC and pSTAT3 is increased in the decidua of patients with PE (134). Furthermore, the proportion of Th1 cells and mature DCs is notably higher in patients with PE, suggesting that upregulation of lnc-DC induces the over-maturation of decidual DCs in PE, leading to an increase in Th1 cells that lead to a persistent inflammatory response (134). Overexpression of lnc-DC in HTR8/SVneo cells inhibits trophoblast invasion and motility by increasing pSTAT3 levels and TIMP1 and 2 expression while decreasing expression of MMP-9, -2 and -3 (135). Thus, lnc-DC regulates trophoblast invasion and motility by modulating STAT3 activation and MMP expression (135).

circRNAs are single-strand ring-like ncRNAs produced by reverse splicing of precursor RNA following transcription and are involved in numerous pathologies including pregnancy complications (136). Expression of circRNA of pregnancy-associated plasma protein A (circPAPPA) is downregulated in PE. Knockdown of circPAPPA in HTR8-S/Vneo cells decreases proliferation and invasion ability. Moreover, miR-384 (which targets STAT3) is a direct target of circPAPPA (57). STAT3 expression is decreased when circPAPPA is knocked down, suggesting that downregulation of circPAPPA facilitates onset and development of PE by suppressing trophoblast invasion and proliferation via modulation of miR-384/STAT3 signaling (Table II) (57).

## 6. Cellular STAT3 modulation in PE

B7-H4 is a type I transmembrane glycoprotein belonging to the B7 family of immune checkpoint proteins, which are involved in regulation of immune response, preventing its excessive activation (137). B7-H4 is normally expressed in the placental villous but its expression significantly decreases in decidua of patients with PE compared with normal controls (138). Contrarily, B7-H4 serum levels are notably increased in patients with PE (139). B7-H4 treatment of SGHPL-5 trophoblast cells inhibits proliferation, migration and invasion while promoting apoptosis by decreasing pAKT, pPI3K and pSTAT3 expression (140). Thus, B7-H4 may serve an important role in shallow trophoblast invasion in PE.

RAR-related orphan receptor A (RORA) is a member of the ROR subfamily that serves as a transcription factor in the regulatory region of ROR-responsive genes; its expression is induced by hypoxia (141,142). It has been reported that RORA expression is increased in PE tissues but also in HTR-8/SVneo cells exposed to hypoxic conditions. Silencing of RORA in HTR-8/SVneo cells increases migration and proliferation while decreasing pSTAT3 and pJAK2 expression. The inhibitory effects of RORA silencing are reversed when cells are treated with the STAT3 activator RO8191 (143). Thus, RORA regulates trophoblast cell proliferation and migration via the JAK2/STAT3 signaling pathway (143).

Basal cell adhesion molecule (BCAM) belongs to the immunoglobulin superfamily and is involved in cellular processes such as cell adhesion, migration and invasion (144). Liu *et al* (58) found that BCAM expression is significantly decreased in PE placenta. Moreover, silencing BCAM in HTR-8/SVneo and JAR cells leads to decreased trophoblast proliferation, migration and invasion and suppresses pSTAT3(Y705) expression through the downregulation of phosphoinositide-3-kinase regulatory subunit 6 (PIK3R6) expression, a kinase that phosphorylates STAT3. However, phosphorylation on S727 of STAT3 is not altered by BCAM deficiency. In addition, adenoviruses containing BCAM short hairpin RNA genes (Ad-shBCAM) cause BCAM deficiency and a PE-like phenotype with elevated systolic blood pressure, proteinuria and FGR. Accordingly, the expression of BCAM, PIK3R6 and pSTAT3 is downregulated in Ad-shBCAM rats (58). Thus, BCAM deficiency decreases trophoblast proliferation, migration and invasion by inhibiting PIK3R6/STAT3 signaling (58).

NADPH oxidase 2 (Nox2) is an important source of ROS and serves a key role in ferroptosis (145,146), a type of programmed cell death due to iron-dependent lipid peroxidation (147). STAT3 serves as an oxidation-responsive transcription factor and plays a key role in regulating ferroptosis as it can bind the promoter region of ferroptosis-associated genes such as glutathione peroxidase 4 (GPX4) (148,149). A previous study (59) found that Nox2 expression is significantly higher in PE placentas while STAT3 and GPX4 expression is decreased. Moreover, STAT3 and GPX4 gene expression is notably decreased by hypoxia and RAS-selective lethal compound 3 (RLS3)-induced ferroptosis while their expression is restored when ferroptosis is inhibited with Ferrostatin-1 (Fer-1). Silencing Nox2 in HTR8/SVneo cells inhibits ferroptosis and increases STAT3 and GPX4 expression (59). Thus, Nox2 may trigger ferroptosis in PE via modulation of the STAT3/GPX4 pathway (59).

NOP2/Sun5 (NSUN5) is an RNA methyltransferase involved in important cell processes such as mitochondria assembly and cell proliferation (150). Zhang *et al* (151) found a notable association between NSUN5 polymorphism (rs77133388) and PE. Pregnant single-base mutant mice (NSUN5 R295C at rs77133388) exhibit PE symptoms and reduced decidualization. Additionally, the aforementioned study found decreased IL-11Rα, cyclin D3, pJAK2 and pSTAT3 expression in NSUN5 R295C mice, suggesting that NSUN5 mutation potentially alters decidualization through IL-11Rα/JAK2/STAT3/cyclin D3 signaling, favouring PE occurrence (151).

Hypoxia-inducible factors (HIFs; HIF-α and HIF-β subunits) are hypoxia-induced transcription factors that regulate cellular processes under hypoxic condition. HIF-1α, HIF-2α and HIF-3α are paralogs of the HIF-α subunit; while HIF-1α and HIF-2α are considered the two master regulators of hypoxic response, little is known about HIF-3α (152). Qu *et al* (153) found that HIF-3α expression is decreased in PE placentas compared with normal pregnancy. Moreover, under chronic hypoxia (72 h), the expression of HIF-3α, pJAK and pSTAT3 is significantly decreased while apoptosis notably increases. Overexpression of HIF-3α in HTR8/SVneo cells markedly increases phosphorylation of JAK/STAT, indicating that HIF-3α upregulation can regulate the JAK/STAT pathway and serves as a protective factor against hypoxia, favoring cell survival (153).

Annexin A1 (ANXA1) is a calcium-dependent phospholipid-binding protein that can bind negatively charged phospholipids and is involved in cell activities such as anti-inflammatory response, differentiation and proliferation, cell signal regulation and phagocytosis of apoptotic cells (154-156). ANXA1 is highly expressed in the plasma of patients with PE (157,158). Feng *et al* (159) found that ANXA1, TNF-α, IL-1β, IL-6 and IL-8 expression are increased in placental tissues of PE rats. In addition, the aforementioned study found that silencing of ANXA1 in trophoblast cells isolated from placentas of PE rat model significantly decreases apoptosis and inflammatory response of trophoblast cells. Furthermore, silencing of ANXA1 significantly increases expression of Bcl-2 and pro-caspase-3, while downregulating the expression of BAX, cleaved-caspase-3, TNF-α, IL-1β, IL-6 and IL-8 (159). Silencing of ANXA1 notably decreases phosphorylation of JAK2 and STAT3 (159). These effects on STAT3 and JAK2 can be explained by an indirect modulation by ANXA1. Decreased phosphorylation of JAK2 and STAT3 may be due to decreased expression of the cytokines TNF-α, IL-1β, IL-6 and IL-8, which activate JAK/STAT3 pathway (160-163). Similar results were obtained by Mo *et al* (164) studying ANXA7, another member of the annexin family (154). Silencing of ANXA7 in HTR-8/SVneo cells induces cell apoptosis and inhibits cell proliferation by downregulating Bcl-2 protein expression (164). Silencing of ANXA7 decreases pJAK and pSTAT3 expression (164). As it has been reported that trophoblast viability is notably decreased when the levels of pJAK and pSTAT3 are reduced (165), the aforementioned results demonstrated that ANXA7 can regulate trophoblast apoptosis by modulating the JAK/STAT3 pathway.

IL-27 is a member of the IL-12/IL-6 family of cytokines produced by antigen-presenting cells and regulates T cell differentiation and function, exerting pro- and anti-inflammatory effects during immune response (166). Expression of IL-27 and its receptor (IL-27 receptor α) is significantly increased in the trophoblast of placentas from PE pregnancy (167). A previous study found that IL-27 significantly inhibits HTR-8/SVneo cell invasion and migration by favouring the expression of epithelial markers over mesenchymal markers. Furthermore, IL-27 induces phosphorylation of STAT1 and STAT3 (168). Silencing of STAT1 attenuates the effects of IL-27, while silencing STAT3 has no effect, demonstrating that IL-27 may inhibit trophoblast cell migration and invasion by affecting epithelial-mesenchymal transition via a STAT1-dominant pathway in PE (168).

Heme oxygenase-1 (HO-1) is a key antioxidant enzyme with anti-hypertensive effects (169) and cytoprotective and anti-inflammatory functions under ischemic conditions (170). HO-1 expression is significantly increased in PE placentas while STAT3 phosphorylation (Y705) is notably decreased. Moreover, human placental choriocarcinoma JEG-3 cells exposed to hypoxia show increased HO-1 expression and STAT3 phosphorylation (Y705) compared with cells cultured under normoxic conditions (60). HO-1 overexpression in JEG-3 cells significantly inhibits hypoxia-promoted STAT3 phosphorylation (Y705), suggesting that the overexpression of HO-1 in PE placentas might contribute to decreased STAT3 phosphorylation (Y705) found in the placentas complicated by PE (60). This is consistent with a study by Xu *et al* (165), reporting an increased expression of pJAK and pSTAT3 in primary third trimester trophoblast cells exposed to hypoxia. To the best of our knowledge, the aforementioned study is the only report of increased expression of total STAT3 expression in PE placentas.

HO-1 expression and activity are induced by cobalt protoporphyrin (CoPP) (171) and HO-1 induction notably attenuates oxidative stress and hypertension in pregnant rats with reduced uterine perfusion pressure (RUPP) (172). A previous study found that phosphorylation of JNK, STAT1, pSTAT3 (Y705) is significantly increased in placental tissues of RUPP rats (65). CoPP decreases RUPP-induced phosphorylation of JNK and STAT1, while increasing phosphorylation of STAT3, indicating that RUPP induces oxidative stress, increasing phosphorylation of mediators of cell death, such as STAT1 and JNK (172,173), and survival, such as STAT3 (174), in placentas of pregnant rats. HO-1 induction by CoPP shifts this balance to a pro-survival phenotype by increasing phosphorylation of the pro-survival STAT3, while suppressing phosphorylation of JNK and STAT1. These results demonstrate therapeutic activity of HO-1 induction in placental cell following ischemic injury (due to the RUPP model) favouring the survival of placental cells. Thus, the HO-1 pathway may be a promising therapeutic target for management of PE (65).

Ribosomal protein S4, Y-linked 1 (RPS4Y1) is a member of the S4E family of ribosomal proteins ubiquitously expressed and is involved in regulation of cell processes such as apoptosis, cell migration and invasion (175,176). A previous study (177) found that RPS4Y1 levels are significantly upregulated in PE placentas. Silencing of RPS4Y1 in HTR8/SVneo cells induces cell invasion and increased STAT3 phosphorylation along with increased expression of N-cadherin and vimentin. These effects are abolished when RPS4Y1 and STAT3 are silenced, demonstrating that RPS4Y1 may be involved in PE, affecting trophoblast cell migration and invasion via the STAT3 pathway (177).

EGF is a polypeptide involved in cell proliferation, differentiation and survival (178). Lower levels of EGF are found in plasma and urine of patients with PE, suggesting a potential role of EGF in this pathology (179). A previous study (180) reported that treatment of HTR-8/SVneo cells with EGF notably increases cell invasion. Moreover, EGF treatment leads to an increase in phosphorylation of ERK1/2, STAT1 and STAT3 (at both Y705 and S727 residues). Inhibition of ERK1/2 phosphorylation by U0126 decreases EGF-mediated invasion and pSTAT3 and pSTAT1 expression. Silencing of STAT3 leads to decreased EGF-mediated invasion of HTR-8/SVneo cells and pSTAT1 expression but does not have any effect on ERK1/2 activation. Silencing of STAT1 also leads to decreased EGF-mediated invasion of HTR-8/SVneo cells and ERK1/2 and STAT3 (at S727 residue) phosphorylation (180). These results suggest crosstalk between ERK1/2 and JAK/STAT pathways during EGF-mediated increase of HTR-8/SVneo cells invasion; phosphorylation at S727 residue of both STAT3 and STAT1 may be critical in this process (180).

Human leucocyte antigen-G (HLA-G) is the primary immune modulator in embryo implantation and allows the interaction between immune cells [such as natural killer (NK) cells] and trophoblast cells, inhibiting NK cytotoxicity and cytokine production (181). Moreover, decreased HLA-G expression may contribute to PE onset (182). Silencing HLA-G in JEG-3 cells decreases invasion capacity but does not alter cell proliferation or apoptosis. Moreover, silencing of HLA-G decreases STAT3 activation, whereas the overexpression of HLA-G promotes STAT3 activation and invasion in JEG-3 cells, demonstrating that HLA-G is able to regulate JEG-3 cell invasion by influencing STAT3 activation explaining implantation defects due to the low HLA-G expression in PE (Table III) (183).

## 7. Others STAT3 modulators in PE

Chorionic villi serve a key role in normal placental development and function, allowing the transport of nutrients and oxygen to the foetus (184). However, the development of chorionic villi is impaired in pregnancy complications such as PE and FGR (18). Chorionic villous mesenchymal stem cells (CV-MSCs) are multipotent cells that are detached from chorionic villi and differentiate *in vitro* into neurocytes and hepatocytes (185). CV-MSCs serve a pivotal role in regulating trophoblast function. Chu *et al* (186) found that treatment of JAR, JEG-3 and HTR-8 cells under hypoxic conditions with CV-MSC supernatant markedly enhances proliferation and invasion and augments autophagy. In addition, pSTAT3 and pJAK2 levels increase following CV-MSC treatment, suggesting that CV-MSC-dependent JAK2/STAT3 signaling activation is a prerequisite for autophagy upregulation in trophoblast cells and an important factor in protecting cells from hypoxia (186).

In addition to CV-MSCs, STAT3 can also be modulated by antiphospholipid antibodies (aPLs), which have been found in patients affected by antiphospholipid syndrome (187,188). aPLs are at risk factor for recurrent miscarriage and PE onset (174) as aPL can bind the β2-glycoprotein I (β2-GPI) expressed by trophoblast cells, triggering an inflammatory response and compromising the invasiveness of trophoblast cells (190). A previous study (191) found that treatment of first trimester trophoblast cells with anti-β2-GPI monoclonal antibodies notably downregulates IL-6 secretion and pSTAT3 expression, reducing trophoblast cell invasion. Thus, aPLs limit trophoblast cell migration by downregulating trophoblast IL-6 secretion and STAT3 activation (191).

## 8. Conclusion

**Figure 4:**
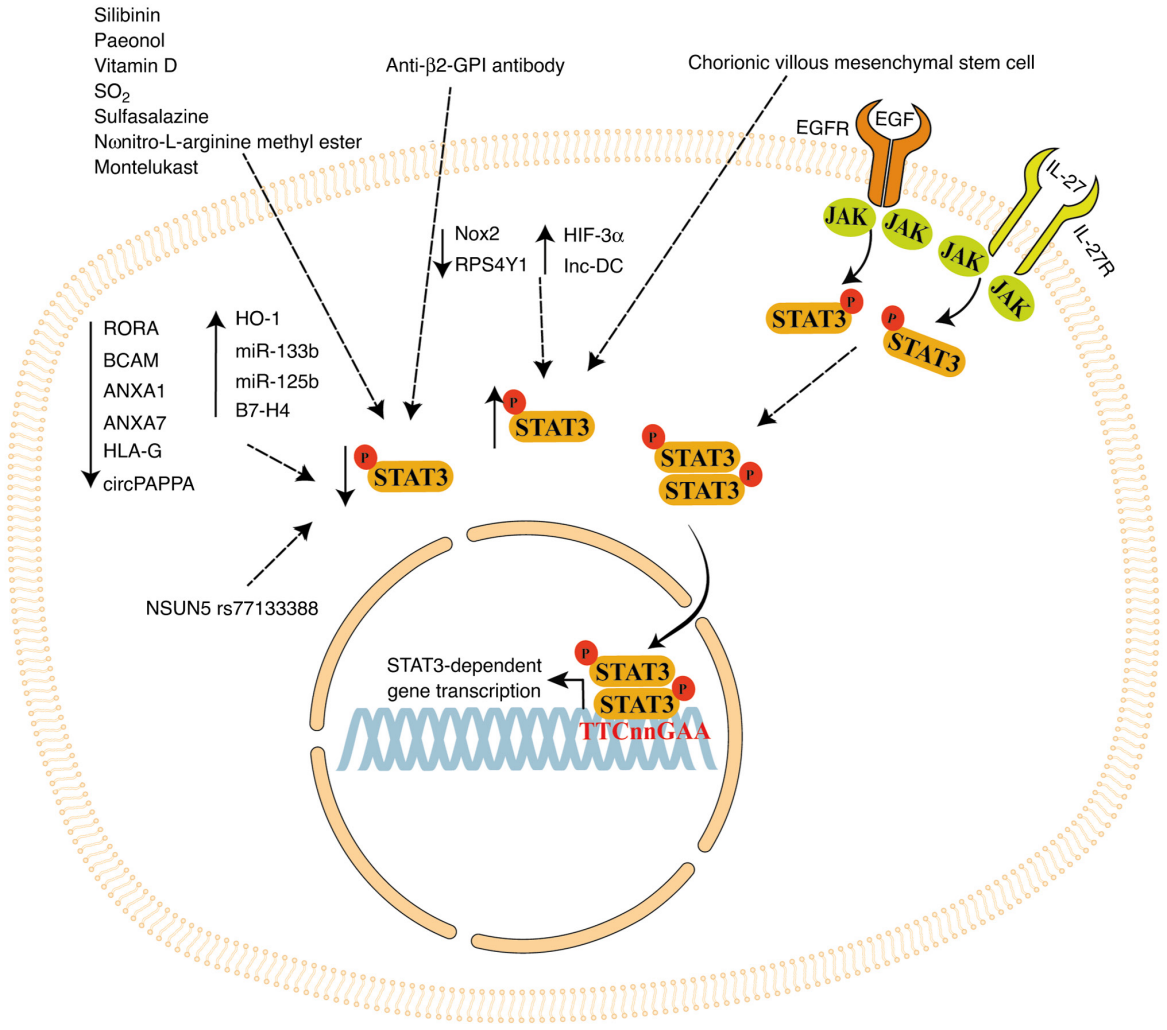
STAT3 signaling regulation by chemical compounds and cellular modulators. pSTAT3 expression is regulated by proteins, cytokines, growth factors, chemical compounds, anti-β2-GPI antibodies, chorionic villous mesenchymal stem cells and non-coding RNAs. GPI, glycosyl phosphatidyl inositol; Nox, NADPH oxidase; HIF, hypoxia-inducible factor; lnc-DC, long non-coding dendritic cells RNA; RPS4Y1, ribosomal protein S4, y-linked 1; HO, heme oxygenase; miR, microRNA; RORA, RAR related orphan receptor A; BCAM, basal cell adhesion molecule; ANXA, annexin A; HLA-G, human leukocyte antigen G; circPAPPA, circular pregnancy-associated plasma protein A RNA; NSUN, Nol1/Nop2/SUN domain.

STAT3 signaling may be a promising target for treatment of PE. The present review summarizes the role of STAT3 signaling in regulating processes in placental cell lines and *in vivo* PE models. STAT3 signaling is regulated by several factors (Fig. 4). In particular, natural compounds such as silibinin, paeonol and vitamin D, as well as synthetic compounds such as SO_2_ derivates, pravastatin, sulfasalazine, L-NAME and montelukast, regulate STAT3 activation and/or expression in inflammatory and trophoblast cells, regulating inflammatory cytokine production and modulating trophoblast cell proliferation and invasion (61,63,64,105,113).

Furthermore, STAT3 expression is modulated by ncRNAs such as miR-133b, miR-125b, lnc-DC and circPAPPA, and cellular modulators such as RORA, BCAM, Nox2, NSUN5, IL-27, HO-1, RPS4Y1, EGF, HLA-G, ANXA1 and ANXA7. Thus, STAT3 signaling plays a key role in important processes for placental development that are impaired in PE placentas. STAT3-dependent alterations in PE may be improved by stimulating the activation of STAT3 signaling. Therapy focused on STAT3 regulation may improve the efficiency of the classical treatments (e.g. magnesium sulfate, heparin) to ameliorate PE outcomes or avoid its onset. Moreover, natural and synthetic compounds decrease pSTAT3 expression (Fig. 4). Use of these compounds must be tightly controlled or avoided since it can significantly worsen STAT3-dependent cellular and molecular processes given that pSTAT3 expression is low in PE placentas (53-55).

As STAT3 expression is notably decreased in PE placentas compared with normal placentas (53,55), its activation (especially in pregnancy at risk of PE development) may promote processes necessary for proper placental development (such as protecting trophoblast cells from apoptosis in a hypoxic environment, such as that at the beginning of placentation).

The role of STAT3 signaling in PE pregnancies has also clinical value. As STAT3 signaling is inhibited by miR-133b, miR-125b, B7-H4 and NSUN5 polymorphism (rs77133388), modulators that can be detected in the blood at first trimester of pregnancy when no PE clinical signs or symptoms are present, increased levels of these modulators in the blood or the presence of rs77133388 could be an indicator of a pregnancy at risk of PE due to a possible impairment of STAT3 signaling.

## Figures and Tables

**Table I:** STAT3 modulation by natural and synthetic compounds.

A, Natural compounds			
Modulator	Model	Results	(Refs.)
Silibinin	PBMC	Decreases STAT3/RORγt, TNF-α, IL-6 and IL-23 levels; increases IL-10 and TGF-β expression	(90)
Paeonol	PE mouse model (induced by PS/PC injection)	Decreases TNF-α, IL-6, and IFN-γ mRNA levels; increases IL-4 mRNA levels; inhibits phosphorylation of JAK2 and STAT3; effects are reversed by treatment with SC-39100, a JAK2/STAT3 pathway agonist.	(62)
Vitamin D	PBMC and CD4^+^ T cells from patients with PE	Decreases STAT1/STAT4/T-bet and STAT3/RORγt signaling and expression of IFNγ, TNFα, IL-17, IL-22, IL-23 and IL-6; increases STAT6/GATA-3 and STAT5/FoxP3 signaling and expression of IL-10 and TGF-β	(97)

B, Synthetic compounds			

Modulator	Model	Results	(Refs.)

SO_2_ derivates	Swan.71 trophoblast cells	Decreases cell migration and invasion; arrests cell cycle at S/G2/M phase; induces apoptosis; increases IL-1β secretion; decreases IL-6 secretion, STAT3 phosphorylation and MMP2 and MMP9 expression	(105)
Pravastatin	PE rat model (induced by deoxycorticosterone acetate injection)	Decreases IL-6, pSTAT1 and pSTAT3 expression	(63)
	PE mouse model (induced by sFlt1 overexpression)	Increases pSTAT3 expression in placenta	(66)
Sulfasalazine	Primary cytotrophoblast cells	Decreases sFlt-1 secretion and protein expression of ERK1/2 and STAT3	(113)
L-NAME	PE rat model (induced by L-NAME)	Decreases sSTAT3 and pSTAT3 expression in the placenta	(61)
Montelukast	PE rat model (induced by L-NAME)	Decreases oxidative stress and expression of IL-6, TNF-α, pJAK2 and STAT3 in placental tissues	(64)

PS/PC, phosphatidylserine/dioleoyl-phosphatidylcholine; L-NAME, Nω-nitro-L-arginine methyl ester; PE, preeclampsia; p, phosphorylated; T-bet, T-box protein.

**Table II:** STAT3 modulation in HTR8/SVneo cells by ncRNAs.

Modulator	Results	(Refs.)
miR-133b	Decreases pJAK2 and pSTAT3 expression and trophoblast migration and invasion; induces apoptosis	(56)
miR-125b	Decreases cell proliferation, invasion and migration and expression of STAT3, pSTAT3 and SOCS3	(129)
lnc-DC	Inhibits cell invasion and motility; increases pSTAT3 and TIMP-1 and -2 expression; decreases MMP-9, -2 and -3 expression	(135)
circPAPPA	Knockdown of circPAPPA decreases cell proliferation and invasion and STAT3 expression by increasing miR-384 expression	(57)

TIMP, tissue inhibitor of metalloproteinase; lnc, long non-coding; miR, microRNA; p, phosphorylated; SOCS, suppressor of cytokine signaling proteins; DC, dendritic cell; circPAPPA, circular pregnancy-associated plasma protein A RNA.

**Table III:** Cellular STAT3 modulation in PE.

Modulator	Model	Results	(Refs.)
B7-H4	SGHPL-5 cells	Inhibits cell proliferation, migration, and invasion; promotesapoptosis; downregulates pPI3K, pAkt and pSTAT3 expression	(140)
RORA	HTR-8 cells	RORA silencing increases cell migration and proliferation and decreases pSTAT3 and pJAK2 expression; STAT3 activator RO8191 reverses the inhibitory effects of RORA silencing	(143)
BCAM	HTR-8/SVneo, JAR cells	BCAM silencing decreases cell proliferation, migration and invasion, as well as pSTAT3(Y705) expression via the downregulation of PIK3R6 but does not alter pSTAT3(S727) expression	(58)
Nox2	HTR-8/SVneo cells and placental tissue	Nox2 expression is increased in PE; Nox2 silencing inhibits ferroptosis and increases mRNA and protein levels of STAT3 and GPX4	(59)
NSUN5	Patients with NSUN5 R295C (rs77133388)	NSUN5 R295C decreases decidualization and IL-11Rα, cyclin D3, pJAK2 and pSTAT3 expression	(151)
HIF-3α	HTR8/SVneo cells	HIF-3α overexpression increases Flt1 expression and phosphorylation of JAK/STAT pathway proteins	(153)
ANXA1	Trophoblast cells isolated from PE rats (induced by L-NAME)	ANXA1, TNF-α, IL-1β, IL-6 and IL-8 expression increases in placental tissue of PE rats; ANXA1 silencing in trophoblast cells increases the expression of Bcl-2 and pro-caspase-3; ANXA1 silencing downregulates the expression of Bcl-2-associated X protein, cleaved-caspase-3, TNF-α, IL-1β, IL-6 and IL-8, as well as phosphorylation of JAK2 and STAT3 without altering their expression	(159)
ANXA7	HTR8/SVneo cells	ANXA7 silencing induces apoptosis, inhibits cell proliferation and decreases phosphorylation of JAK and STAT3(Y705) without altering their expression	(164)
IL-27	HTR8/SVneo cells	IL-27 inhibits HTR-8/SVneo cells invasion and migration and induces phosphorylation of STAT1 and STAT3; STAT1 silencing attenuates the effect of IL-27, while silencing STAT3 has no effect	(168)
HO-1	PE placenta and JEG-3 cells	Hypoxia increases HO-1 and pSTAT3(Y705) expression; HO-1 overexpression in JEG-3 cells inhibits hypoxia-promoted pSTAT3(Y705) expression	(60)
HO-1	PE mouse model (induced by RUPP)	HO-1 inducer cobalt protoporphyrin increases pSTAT3 (Y705) expression	(65)
RPS4Y1	HTR8/SVneo cells	RPS4Y1 silencing induces cell invasion and increasespSTAT3(Y705), N-cadherin and vimentin expression; these effects are abolished when RPS4Y1 and STAT3 are silenced	(177)
EGF	HTR8/SVneo cells	EGF increases phosphorylation of ERK1/2, STAT1 (S727) and STAT3 (at both Y705 and S727 residues); inhibition of ERK1/2 phosphorylation by U0126 decreases EGF-mediated invasion and pSTAT3 and pSTAT1 expression; STAT3 silencing decreases EGF-mediated invasion and pSTAT1 expression but does not have any effect on ERK1/2 activation; STAT1 silencing decreases EGF-mediated invasion and ERK1/2 and STAT3 (at S727 residue) phosphorylation	(180)
HLA-G	JEG-3 cells	HLA-G silencing decreases STAT3 activation; HLA-G overexpression promotes STAT3 activation and cell invasion	(183)

RORA, RAR-related orphan receptor A; BCAM, basal cell adhesion molecule; Nox2, NADPH oxidase 2; ANXA1, annexin A1; HO-1, heme oxygenase 1; EGF, epidermal growth factor; HLA-G, human leucocyte antigen-G; RUPP, reduced uterine perfusion pressure; PE, preeclampsia; p, phosphorylated; Flt, fms-like tyrosine kinase; L-NAME, Nω-nitro-L-arginine methyl ester; RPS4Y1, ribosomal protein S4, y-linked 1; NSUN, Nol1/Nop2/SUN domain.

## Data Availability

Not applicable.
